# Systematic investigation of the diffraction efficiency of sub-15 nm resolution soft X-ray zone plates via simulation and measurement

**DOI:** 10.1107/S160057752600860X

**Published:** 2026-09-18

**Authors:** Qingyuan Mao, Yuchen Jiao, Liang Liu, Jingyuan Zhu, Li Jiang, Xiangzhi Zhang, Zhanshan Wang

**Affiliations:** a Institute of Precision Optical Engineering, School of Physics Science and Engineering Tongji University Shanghai200092 People’s Republic of China; b MOE Key Laboratory of Advanced Micro-Structured Materials, Shanghai200092, People’s Republic of China; c Shanghai Frontiers Science Center of Digital Optics, Shanghai200092, People’s Republic of China; d Shanghai Institute of Applied Physics Chinese Academy of Sciences Shanghai201800 People’s Republic of China; e University of Chinese Academy of Sciences Shanghai100049 People’s Republic of China; f Shanghai Synchrotron Radiation Facility, Shanghai Advanced Research Institute Chinese Academy of Sciences Shanghai201204 People’s Republic of China; g Shanghai Institute of Intelligent Science and Technology Tongji University Shanghai200092 People’s Republic of China; Forschungszentrum Jülich, Germany

**Keywords:** Fresnel zone plates, diffraction efficiency, soft X-ray microscopy, rigorous coupled-wave analysis

## Abstract

Soft X-ray Fresnel zone plates (FZPs) are core components in synchrotron radiation microscopy systems, and their diffraction efficiency directly determines focal intensity and imaging performance. Currently, research on 15 nm high-resolution FZPs has been primarily focused on spatial resolution, while experimental investigations into their diffraction efficiency remain scarce. Moreover, inconsistencies observed among different measurement approaches significantly hinder the accurate evaluation of their optical performance. In this study, we conducted systematic comparative measurements of the relative and absolute diffraction efficiencies of an FZP with a 12.5 nm outermost zone width. By integrating the beamline’s active closed-loop vibration suppression and high-speed real-time incident flux normalization, we effectively mitigated environmental noise and top-up injection fluctuations. This specific setup enabled highly repeatable efficiency measurements over the short continuous measurement period at the 15 nm resolution limit, yielding a relative RMS fluctuation below 3%. These measurement results were subsequently benchmarked against a whole-aperture, area-averaged rigorous coupled-wave analysis model. Results indicate that relative diffraction efficiency aligns well with the theoretical calculations, supporting the overall consistency between the measured and calculated energy responses. Conversely, absolute diffraction efficiency more directly quantifies the realized first-order energy conversion under the present synchrotron conditions by accounting for practical factors, including membrane transmission loss, alignment errors, and incident intensity fluctuations. This precise characterization and systematic analysis of the FZP with a 12.5 nm outermost zone width provide a useful reference for evaluating high-resolution optics and support the optimized design and standardized development of next-generation synchrotron beamlines.

## Abstract 2

This study presents systematic measurements and rigorous coupled-wave analysis of relative and absolute diffraction efficiencies for sub-15 nm soft X-ray Fresnel zone plates. By decoupling intrinsic optical performance from practical beamline transmission losses, this work establishes a highly reliable metrology framework to support the development of next-generation high-resolution synchrotron optics.

## 1. Introduction

Soft X-ray microscopy and spectroscopy at modern synchrotron radiation facilities serve as vital tools for investigating the nanostructures of materials and biological samples (Dyhr *et al.*, 2023 ▸; Aidukas *et al.*, 2024 ▸; Weinhardt & Larabell, 2025 ▸). As the core optical elements in these systems, Fresnel zone plates (FZPs) fundamentally determine the imaging capabilities. The outermost zone width of an FZP dictates its spatial resolution (Chao *et al.*, 2005 ▸, 2012 ▸; Rösner *et al.*, 2020 ▸), while its diffraction efficiency governs the focused beam intensity and overall imaging efficiency (Reinspach *et al.*, 2011 ▸; Tong *et al.*, 2024 ▸). However, current FZP research focuses predominantly on pushing spatial resolution limits, while detailed accounts of diffraction efficiency—both in simulation and experiment—remain scarce. Methods variations in these simulations and experiment measures further lead to inconsistent measured diffraction efficiency data across different studies. Furthermore, a substantial gap often persists between experimental measurements and their theoretical counterparts (Engelberg & Levy, 2022 ▸; Menon & Sensale-Rodriguez, 2023 ▸).

Theoretical frameworks for evaluating the diffraction efficiency of FZPs have become well established. Early work by J. Kirz utilized the thin-grating approximation to define the initial theoretical boundaries for phase-shifting FZPs (Kirz, 1974 ▸). As spatial resolution and efficiency improved, coupled-wave theory was introduced to account for volume effects (Maser & Schmahl, 1992 ▸), followed by the development of rigorous coupled-wave analysis (RCWA) (Gaylord & Moharam, 1985 ▸; Moharam *et al.*, 1995 ▸) and the beam propagation method (BPM) for analyzing complex diffraction fields and multiple diffraction orders (Kurokhtin & Popov, 2002 ▸; Srisungsitthisunti *et al.*, 2009 ▸; Tong *et al.*, 2022 ▸). These sophisticated simulations now enable high-precision predictions of the ultimate optical characteristics of FZPs. On the experimental side, numerous studies have reported diffraction efficiency measurements for state-of-the-art FZPs. For instance, J. Vila-Comamala *et al.* tested iridium (Ir)-based FZPs with a 15 nm outermost zone width at the ESRF ID06 beamline (Vila-Comamala *et al.*, 2011 ▸). J. Reinspach *et al.* characterized Ni–Ge FZPs with 13 nm resolution (Reinspach *et al.*, 2011 ▸), while K. Li *et al.* measured the absolute diffraction efficiency of 16 nm FZPs fabricated via the MACE-ALD process at the NSLS-II Hard X-ray Nanoprobe beamline (Li *et al.*, 2020 ▸). Despite these advancements, a significant discrepancy remains between experimental results and theoretical predictions in nearly all cases. While these gaps are commonly attributed to fabrication defects—such as duty-cycle inaccuracies or the influence of supporting structures—the fundamental differences between testing methodologies are frequently overlooked (Chen *et al.*, 2020 ▸; Yurgens *et al.*, 2020 ▸). Current characterization predominantly follows two distinct paths: relative and absolute diffraction efficiency measurements. The lack of systematic comparison and error analysis between these two approaches makes it difficult for researchers to decouple whether efficiency losses stem from intrinsic fabrication flaws or extrinsic measurement errors. This ambiguity in characterization standards significantly limits the objective performance evaluation of high-resolution diffractive optics.

This study utilizes the advanced soft X-ray experimental platform at the Shanghai Synchrotron Radiation Facility (SSRF) to evaluate high-resolution FZPs with a 12.5 nm outermost zone width. We implement a comprehensive evaluation framework that integrates theoretical simulations with both relative and absolute diffraction efficiency measurements. To overcome the inherent instabilities associated with extreme weak-signal detection at the sub-15 nm limit, we incorporated the active closed-loop vibration suppression and high-speed real-time incident flux normalization systems of the beamline (Yao *et al.*, 2024 ▸; Jiao *et al.*, 2025 ▸). This setup effectively mitigates environmental mechanical noise and periodic source fluctuations, achieving highly reproducible diffraction efficiency measurements with a relative RMS fluctuation below 3%. A detailed comparative analysis between the dual-path experimental results and RCWA simulations clarifies the physical significance and practical applications of each measurement approach. Our results demonstrate that relative diffraction efficiency largely removes the influence of attenuation by the matched supporting membrane and serves as a useful benchmark for model–experiment comparison. In contrast, absolute diffraction efficiency accounts for engineering factors—such as membrane absorption, beamline alignment, and incident intensity fluctuations—providing a realistic assessment of energy conversion under operational synchrotron conditions. This work not only establishes a reliable experimental basis for the precision characterization of 15 nm resolution-scale FZPs but also provides critical support for the design of next-generation high-resolution synchrotron beamlines and standardized device development.

## 2. Design, simulation and fabrication of 15 nm resolution FZP

### 2.1. FZP design

To systematically conduct a comparative study of diffraction efficiency, we first designed and fabricated an ultra-high-resolution soft X-ray FZP. The device fabrication is based on the zone-doubling technique proposed by K. Jefimovs *et al.* (Jefimovs *et al.*, 2007 ▸). Microscopically, a hydrogen silsesquioxane (HSQ) resist template in the outermost region was patterned with a 12.5 nm linewidth and a 37.5 nm gap. The thickness of the HSQ was set to 80 nm. Subsequently, atomic layer deposition (ALD) was employed to conformally grow a 12.5 nm-thick platinum (Pt) layer over the template, thereby achieving spatial frequency doubling at the outermost area. The FZP was designed to precisely match the beam acceptance angle and flux requirements of the BL08U1A soft X-ray beamline at SSRF. The overall effective diameter is 300 µm, featuring an 80 µm-diameter central beam stop. This beam stop is specifically paired with a 70 µm-diameter order sorting aperture (OSA) at the beamline to effectively block zeroth-order diffraction. Based on this design, the innermost zone width of the FZP is approximately 55.6 nm, and the outermost feature linewidth reaches 12.5 nm.

### 2.2. RCWA simulation

To model the diffraction efficiency of the Pt/HSQ FZP with a 12.5 nm outermost zone width while accounting for its finite 80 nm structure height, material absorption, and energy-dependent complex refractive indices, we employ RCWA within a local grating approximation framework (Sheng *et al.*, 1997 ▸). The core concept is that, given the minimal variation in feature size between adjacent zones, the entire FZP can be radially discretized into multiple local periodic gratings. The overall FZP efficiency is then obtained by calculating the first-order diffraction efficiency of each local grating via RCWA and combining them by approximately equal-area averaging. To ensure computational accuracy, the optical constants of Pt and the HSQ resist were sourced from the CXRO database, and the structural thickness was set to 80 nm. The RCWA calculations were implemented using Reticolo_V9, with the Fourier truncation parameter set to nn = 50, corresponding to 101 retained Fourier harmonics; this setting was confirmed to be sufficient for convergence in the present first-order efficiency calculation. It is crucial to note that the central Au beam stop is not a perfect light-blocking layer. To ensure the ultimate rigor of our theoretical benchmarking, this energy-dependent residual transmission was quantitatively evaluated using the material’s attenuation length and explicitly incorporated into the global RCWA integration model to correct the effective background flux.

**Figure 1:**
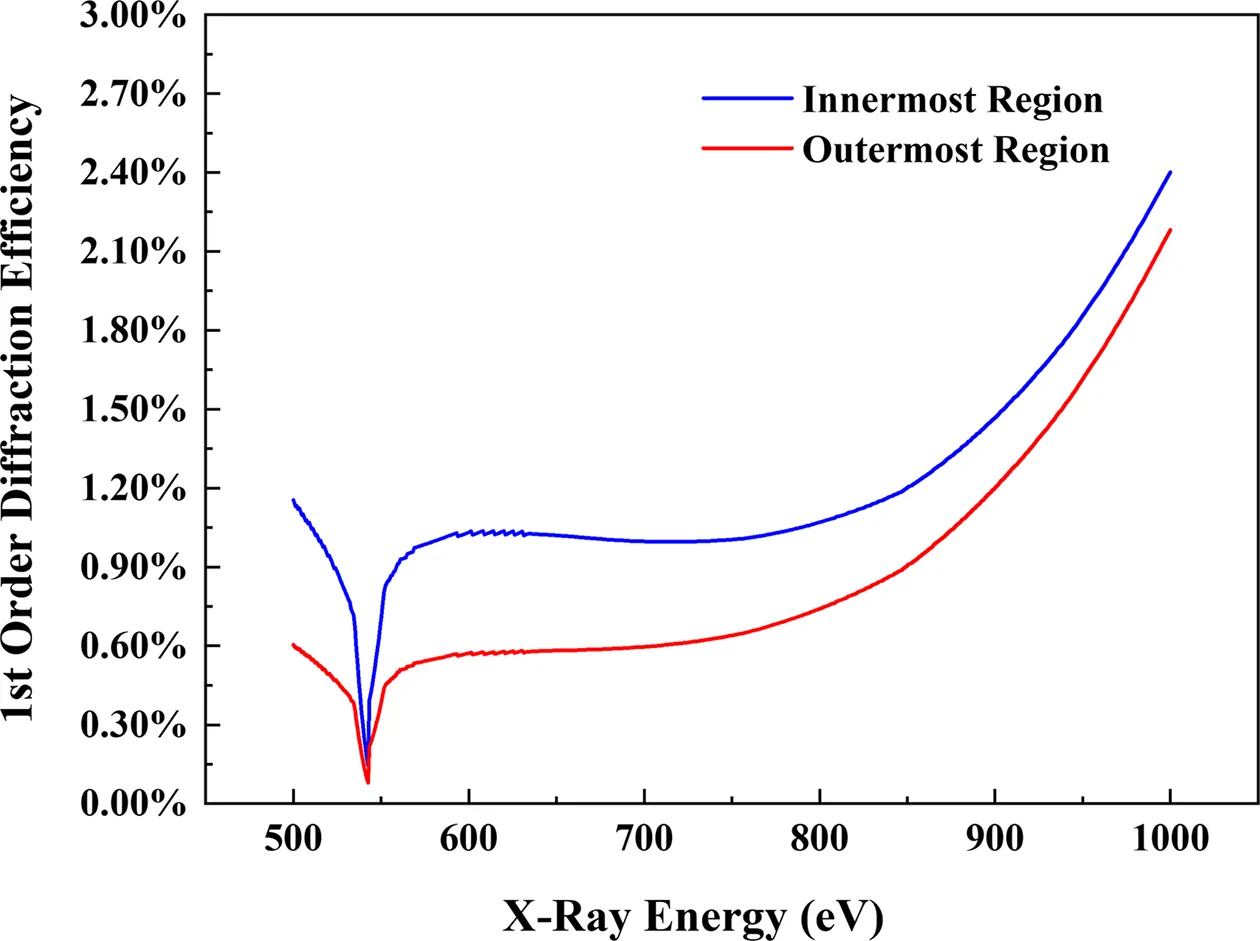
Calculated first-order diffraction efficiency of the outermost zone region and the innermost zone region of the zone plate in the 500–1000 eV X-ray energy.

**Figure 2:**
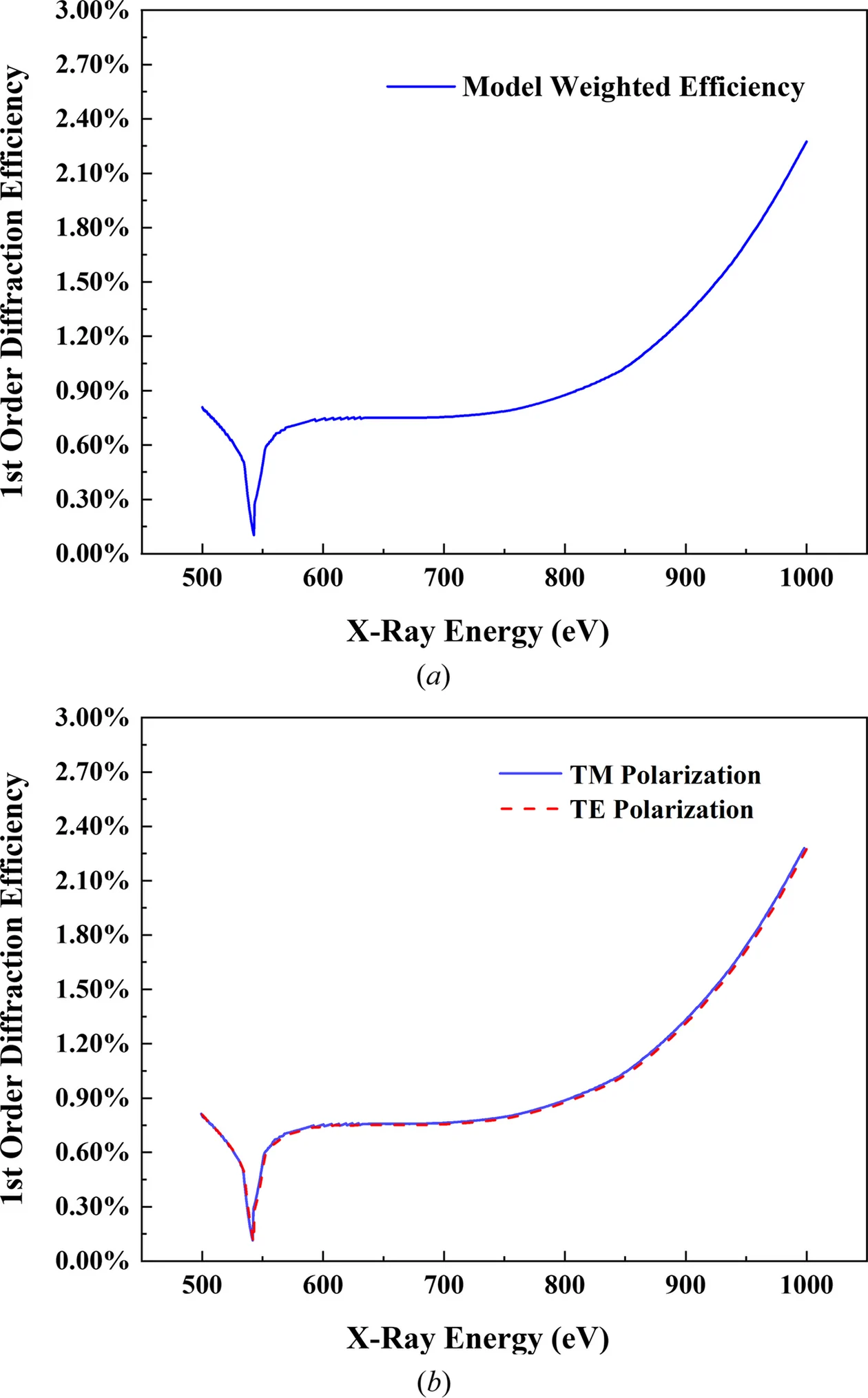
(*a*) RCWA-calculated first-order diffraction efficiency curve of the zone plate based on the local grating approximation model. (*b*) RCWA-calculated first-order diffraction efficiency of the zone plate under TE and TM polarizations.

To evaluate the impact of local period variations on the optical properties, we first independently modeled the two extreme radial regions: the outermost zone (a periodic structure of 12.5 nm Pt / 12.5 nm HSQ / 12.5 nm Pt / 12.5 nm air) and the innermost zone (a periodic structure of 12.5 nm Pt / 55.6 nm HSQ / 12.5 nm Pt / 55.6 nm air). As shown in Fig. 1 ▸, despite efficiency differences arising from minor duty cycle and pitch variations, the inner and outer zones maintain highly consistent overall energy response trends. This validates the rationale behind the localized discretization. Based on this assessment, the FZP was divided into 100 approximately equal-area annular regions for the local grating approximation calculation, with annular boundaries aligned to the nearest actual zone-period boundaries. This model systematically predicts the theoretical diffraction efficiency of the FZP across the 500–1000 eV soft X-ray range, as plotted in Fig. 2 ▸. Furthermore, considering the potential anisotropy induced by the ultra-fine linewidth and high aspect ratio, we evaluated the effect of the incident polarization state. Initial vector diffraction calculations show that at this soft X-ray energy range and specific thickness the efficiency difference between transverse-electric (TE) and transverse-magnetic (TM) polarizations is negligible. This indicates that polarization-dependent perturbations on the overall device efficiency are essentially negligible. It further ensures the reliability of our RCWA model in matching the linearly polarized illumination typical of synchrotron beamlines.

### 2.3. Fabrication

**Figure 3:**
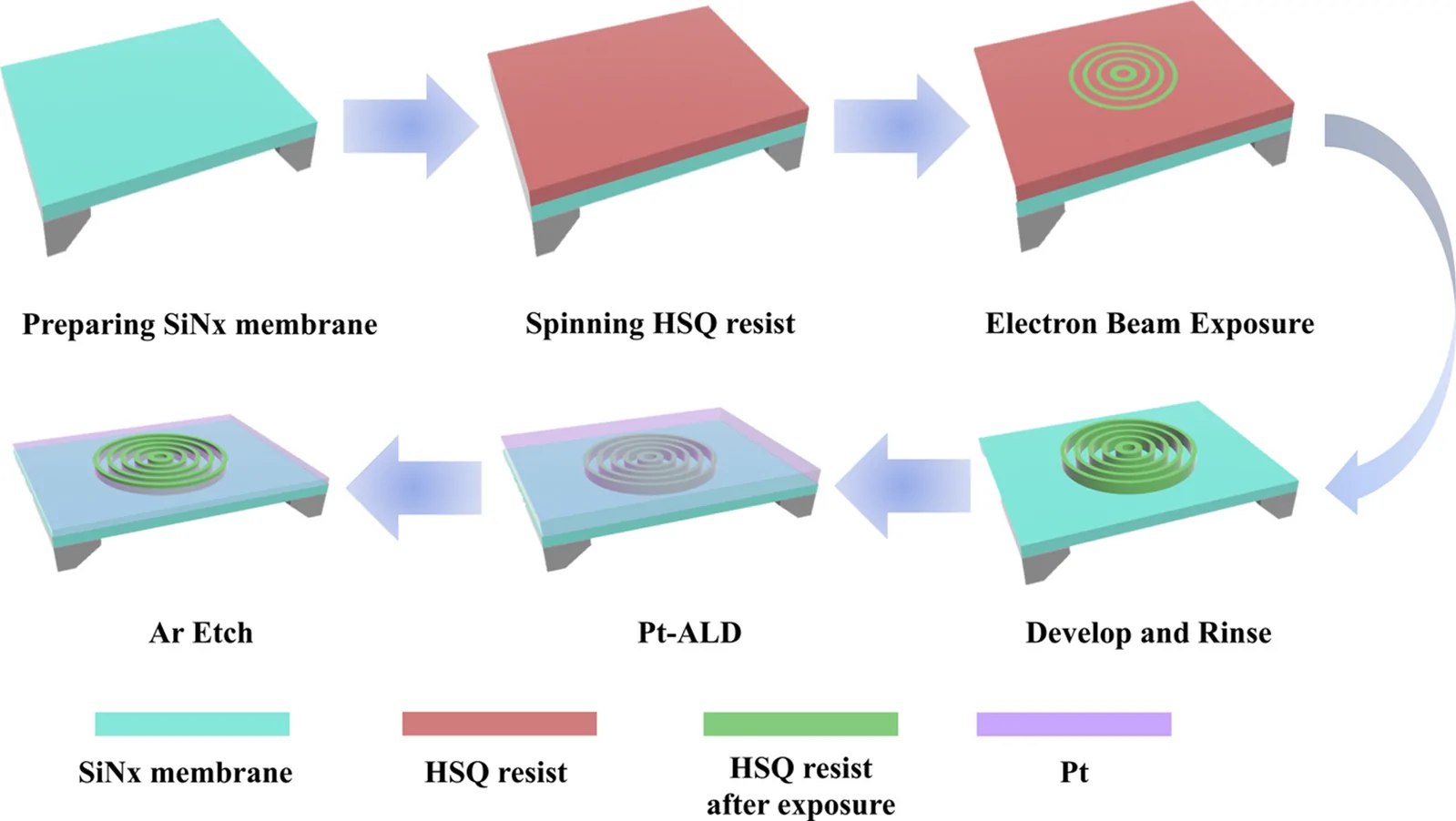
Soft X-ray zone plate fabrication process flow.

**Figure 4:**
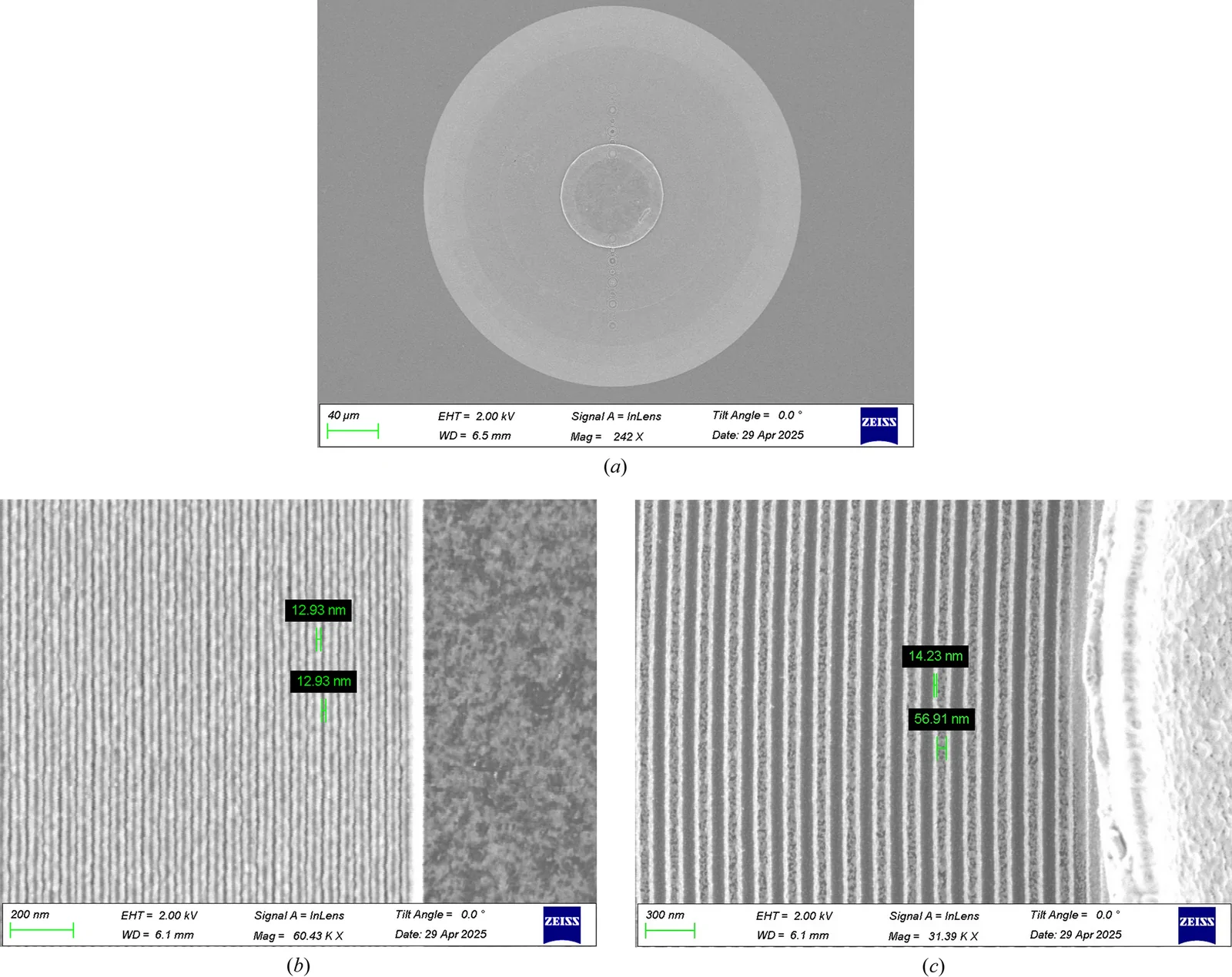
Scanning electron microscopy (SEM) images of the soft X-ray zone plate: (*a*) overall view, (*b*) outermost region, and (*c*) innermost region.

Using optimized high-resolution electron beam lithography (EBL) and atomic layer deposition (ALD) processes, we fabricated four soft X-ray FZPs with nominally identical designs and fabrication parameters in the same batch, each having a 12.5 nm outermost zone width. The nanofabrication workflow is illustrated in Fig. 3 ▸. Initially, a 10 nm Cr adhesion layer and a 15 nm Au seed layer were sequentially deposited onto a 100 nm-thick low-stress silicon nitride (Si_3_N_4_) window. This surface modification enhances the uniformity of the subsequently deposited Pt layer during ALD. The intrinsic soft X-ray absorption loss of this multilayer supporting membrane directly affects the subsequent absolute diffraction efficiency measurements. Conversely, when calculating the relative diffraction efficiency to assess the intrinsic optical performance of the device, this loss is subtracted as background attenuation. Following substrate preparation, MIBK-diluted FOx-16 negative HSQ electron beam resist was spin-coated to form a uniform 80 nm-thick layer. Direct-write exposure of the FZP pattern was then performed using a 100 kV electron beam lithography system. To mitigate the proximity effect between dense nano-zones, we applied gradient exposure doses based on the specific linewidths at different radial positions (Mao *et al.*, 2025 ▸). This ensures high pattern fidelity across all zone regions. After exposure, the sample was developed in a 2.38% TMAH aqueous solution at a constant temperature of 45°C for 240 s. This high-temperature development enhances the contrast of the ultra-fine structures. The sample was then thoroughly rinsed with deionized water and gently blown dry with high-purity nitro­gen. During the zone structure formation phase, a 12.5 nm-thick Pt layer was grown over the HSQ template at 200°C, using (tri­methyl)­methyl­cyclo­pentadienylplatinum as the ALD precursor. Subsequently, argon ion beam etching was employed to remove the redundant Pt layer from the top of the structures, completing the spatial frequency doubling in the outermost FZP region. Finally, a central Au beam stop with an 80 µm diameter and a thickness of approximately 2 µm was fabricated to fully block the incident zeroth-order light. Scanning electron microscopy (SEM) characterization of the fabricated high-resolution FZP is presented in Fig. 4 ▸. The images reveal that both the inner and outer zones maintain excellent structural integrity under this comprehensive fabrication protocol. The transferred 12.5 nm Pt zone structures exhibit a duty cycle closely approaching the target 1:1 ratio. Furthermore, no obvious zone collapse or adhesion is observed, despite the high aspect ratio exceeding 6:1.

## 3. Measurement of diffraction efficiency

**Figure 5:**
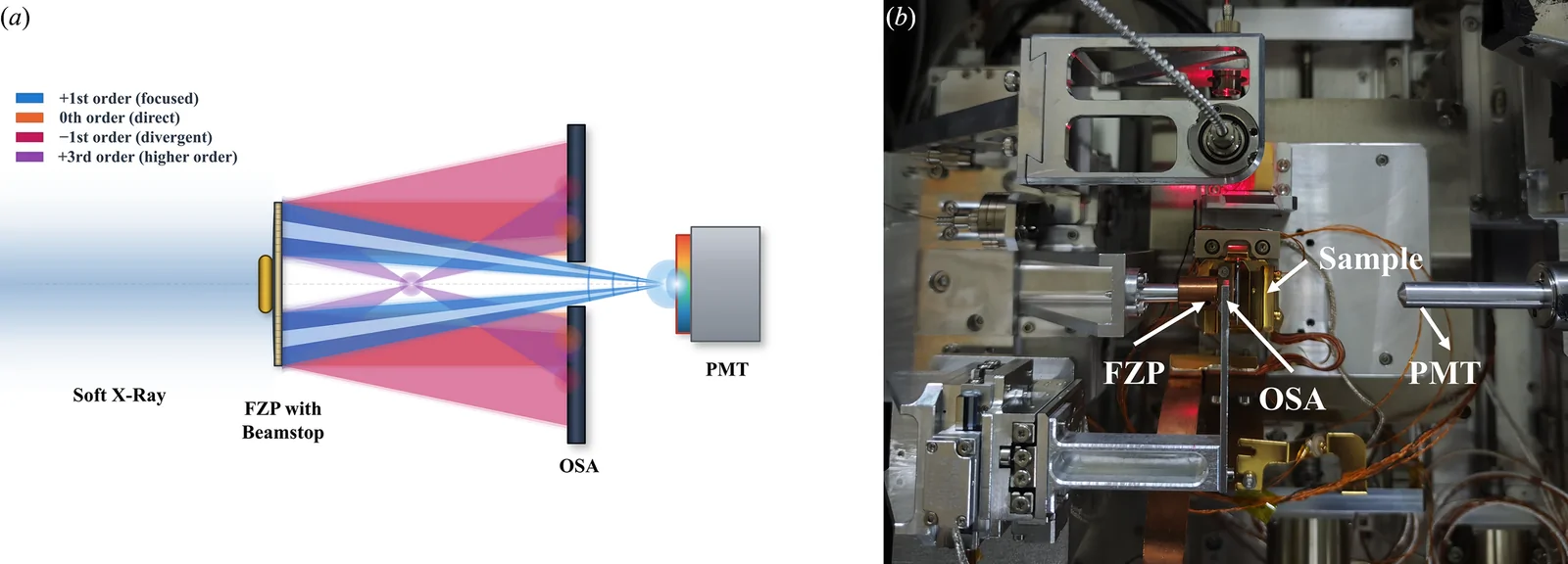
(*a*) Schematic of the optical layout for soft X-ray imaging and diffraction-efficiency measurements. The positive first-order (+1) diffracted beam passes through the OSA, whereas the zeroth-order direct beam, the divergent negative first-order (−1) diffracted beam, and the representative positive third-order (+3) diffracted beam are intercepted by the OSA. The optical paths are shown schematically and are not to scale. (*b*) Photograph of the actual experimental setup at SSRF beamline BL08U1A, with arrows indicating the positions of the FZP, OSA, and PMT detector.

One of these four nominally identical FZPs was selected for synchrotron imaging and diffraction-efficiency characterization at the soft X-ray microscopy beamline BL08U1A of the SSRF; all optical results reported below refer to this tested FZP. At the FZP sample plane, the incident beam size was approximately 400 µm, fully covering the 300 µm-diameter FZP; the monochromator exit slit was 50 µm × 50 µm, corresponding to a resolving power of *E*/Δ*E* = 6440 under the present beamline setting. The undulator beam, modulated by a monochromator, provided X-ray illumination with excellent monochromaticity and spatial coherence. As illustrated in the experimental setup in Fig. 5 ▸, the FZP sample was mounted on a high-precision multi-axis scanning stage for accurate three-dimensional positioning and rotational adjustment. To purify the diffraction signal, a spatial filtering architecture combining a central beam stop and a downstream OSA (70 µm in diameter) was employed. This configuration effectively blocks the direct zeroth-order light and other higher-order diffraction background noise, ensuring extreme order purity of the flux entering the downstream detection system. An independent beam intensity monitoring system (BIMS) was used to monitor and normalize temporal fluctuations in the incident X-ray intensity in real time, while the ultra-weak transmitted X-ray signals were collected with high sensitivity by a photomultiplier tube (PMT).

Prior to the quantitative diffraction efficiency metrology, the intrinsic imaging performance of the FZP was evaluated to verify its optical focusing capability in a real-world synchrotron environment. The resolution test was performed in scanning transmission X-ray microscopy (STXM) mode at 870 eV. In this measurement, the first-order focused beam from the FZP illuminated the test target, and the target was raster-scanned at the focal plane. The transmitted signal was recorded pixel by pixel using a PMT. The tested FZP had an effective diameter of 300 µm and a measured focal length of approximately 2750 µm at 870 eV, corresponding to a nominal numerical aperture of approximately 0.055. The incident beam diameter at the FZP plane was approximately 400 µm, larger than the effective FZP diameter, so that the full aperture of the FZP, including the outermost zones, was illuminated. The STXM image was acquired over a 2 µm × 2 µm field of view with a 5 nm scan step (corresponding to an effective pixel size of 5 nm × 5 nm) and a 1 ms pixel integration time.

**Figure 6:**
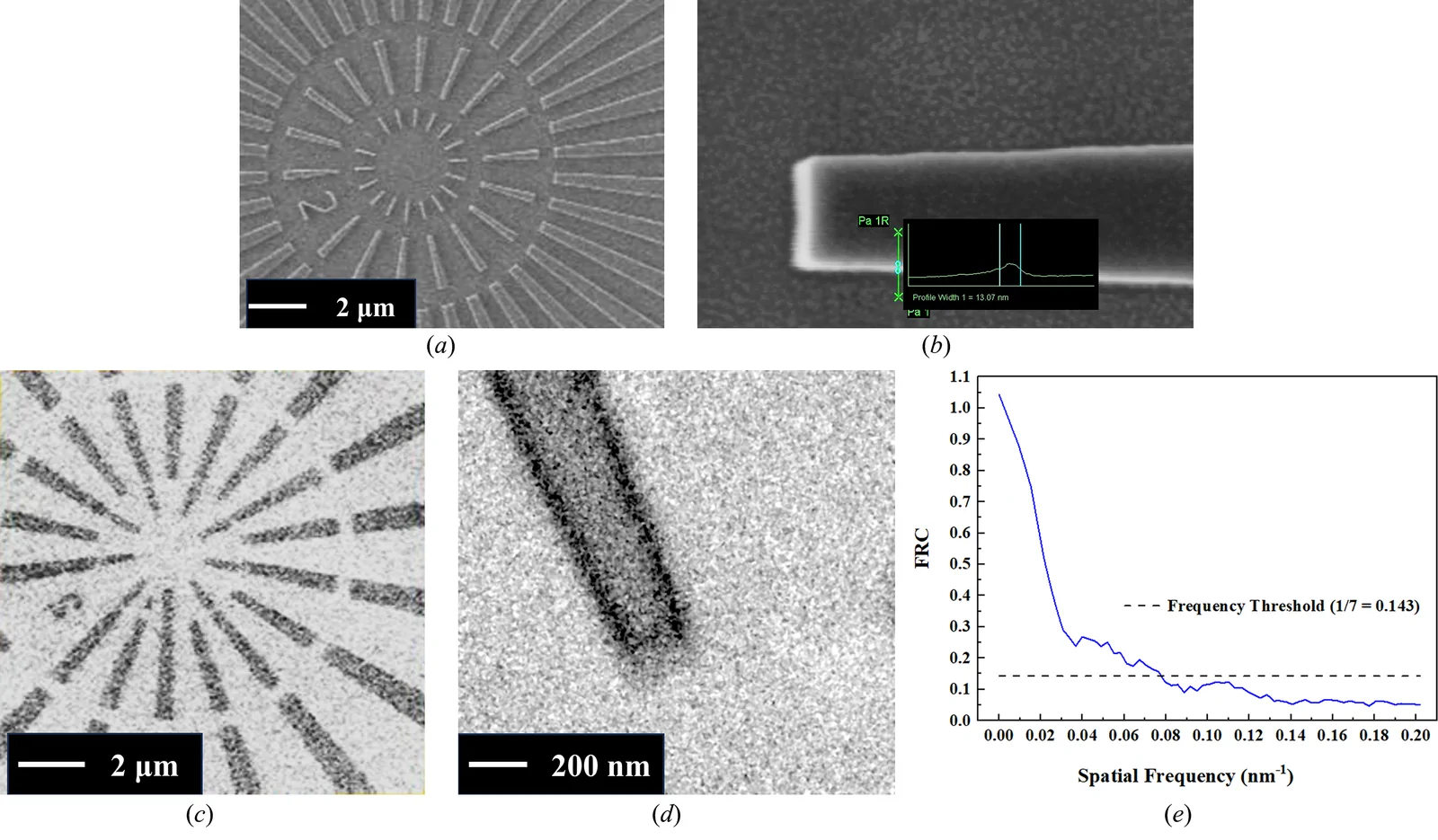
Resolution test results of the sub-15 nm soft X-ray zone plate. The resolution test was performed in STXM mode at 870 eV. (*a*) Overview SEM image of the Siemens star resolution test target. (*b*) Local SEM image of the resolution test target; the inset shows the SEM line profile across the marked Pt-coated edge, corresponding to an apparent edge width of approximately 13.1 nm. (*c*) Overall soft X-ray STXM image of the resolution test target. (*d*) Local soft X-ray STXM image of the resolution test target; this panel shows one representative input image, and the Pt-defined target edges are clearly distinguished from the adjacent target structures. (*e*) FRC analysis based on two independently acquired STXM images of the same local region. The dashed horizontal line denotes the 1/7 FRC threshold (0.143). Its intersection with the FRC curve at *k*τ = 0.077 nm^−1^ corresponds to an FRC-estimated full-period spatial resolution of 13.0 nm.

A Siemens star resolution test target was used for this evaluation. The target consisted of an approximately 300 nm-thick HSQ structure fabricated by electron-beam lithography, with an approximately 13 nm-thick Pt layer deposited on its surface by atomic layer deposition. At 870 eV, the Pt-coated edges provided clear absorption contrast with respect to the adjacent HSQ structure and the surrounding region. The overall resolution-test results are shown in Fig. 6 ▸. Fig. 6 ▸(*a*) shows an overview SEM image of the resolution test target, and Fig. 6 ▸(*b*) shows a local SEM image of the target. The inset in Fig. 6 ▸(*b*) shows the SEM line profile across the marked Pt-coated edge, corresponding to an apparent edge width of approximately 13.1 nm. Fig. 6 ▸(*c*) shows the overall soft X-ray STXM image of the resolution test target, and Fig. 6 ▸(*d*) shows a local soft X-ray STXM image of the target. In the local soft X-ray image, the Pt-defined target edges are clearly distinguished from the adjacent target structures.

To reduce the subjectivity of visual inspection, the image resolution was further estimated using Fourier ring correlation (FRC). In this method, two STXM images independently acquired from the same test region are transformed into Fourier space to obtain the complex spectra F_1_ and F_2_. Fourier space is then divided into concentric rings according to radial spatial frequency. For each frequency ring *s*, the normalized correlation coefficient between the two Fourier spectra is calculated as
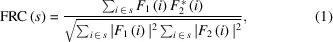
where 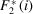 is the complex conjugate of *F*_2_(*i*), and the summation is performed over all Fourier pixels *i* on frequency ring *s*. The FRC threshold used in this work was the commonly applied 1/7 criterion,
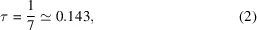

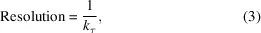
where *k*_τ_ is the spatial frequency at which the FRC curve intersects the 1/7 threshold. The reported value corresponds to the full-period spatial resolution defined by the FRC threshold intersection. For the representative result shown in Fig. 6 ▸(*e*), *k*_τ_ = 0.077 nm^−1^, corresponding to an FRC-estimated full-period spatial resolution of 13.0 nm. These results support sub-15 nm imaging performance of the FZP under the stated STXM conditions.

Following the confirmation of the optical focusing performance, we systematically performed the quantitative diffraction-efficiency measurements. The absolute diffraction efficiency, η_abs_, is defined as the ratio of the first-order focused diffraction signal *I*_1_ detected at the focal plane to the reference incident signal *I*_0_,
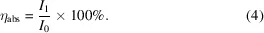
In this definition, *I*_0_ was measured using a 300 µm silicon aperture that matched the outer diameter of the FZP. During the *I*_0_ measurement, the FZP and OSA were removed, while an 80 µm circular silicon stop was placed at the FZP position to geometrically match the central beamstop. Thus, the experimental efficiency was normalized to the same aperture and central-obstruction geometry used in the whole-aperture RCWA comparison.

Notably, at the sub-15 nm spatial-resolution scale, the first-order focal signal is relatively weak, and the measured first-order diffraction efficiency can therefore be affected by minute beam drifts or environmental vibrations. To reduce measurement uncertainty caused by the intrinsic intensity fluctuations of the synchrotron source, all acquired PMT signals were normalized in real time using the independent BIMS monitoring data, and the system dark current was subtracted. For the diffraction-efficiency measurements, we used an integration time of 20 ms and continuously acquired ten repeated sets of *I*_1_ and *I*_0_ data for statistical averaging. Under this detection setting, the nominal linear count-rate limit of the photon-counting system in the ÷10 prescaling mode was 1.0 × 10^7^ s^−1^, and the maximum count rates recorded for *I*_0_ and *I*_1_ were approximately 5.0 × 10^6^ s^−1^ and 7.5 × 10^4^ s^−1^, respectively, both within this linear response range. The results showed good repeatability across these ten repeated datasets. This repeatability reduced the influence of random noise on ultra-weak signal detection and improved the reliability of the diffraction-efficiency measurements under the present beamline conditions.

Furthermore, to accurately assess the intrinsic optical conversion capability of the FZP structure itself, it is necessary to measure the relative diffraction efficiency, η_rel_, thereby excluding the absorption loss introduced by the multilayer supporting membrane. We inserted a blank substrate, identical to the FZP sample substrate, into the beam path. This substrate similarly consists of a 100 nm-thick silicon nitride membrane coated sequentially with a 10 nm Cr adhesion layer and a 15 nm Au seed layer. The detector then recorded the transmitted signal passing through this complete supporting membrane, denoted as the relative transmitted intensity 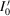. The relative diffraction efficiency is thus defined as the ratio of the first-order diffraction signal *I*_1_ to the incident X-ray signal 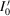 attenuated by the identical supporting substrate,
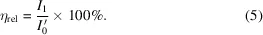


## 4. Results and discussion

**Figure 7:**
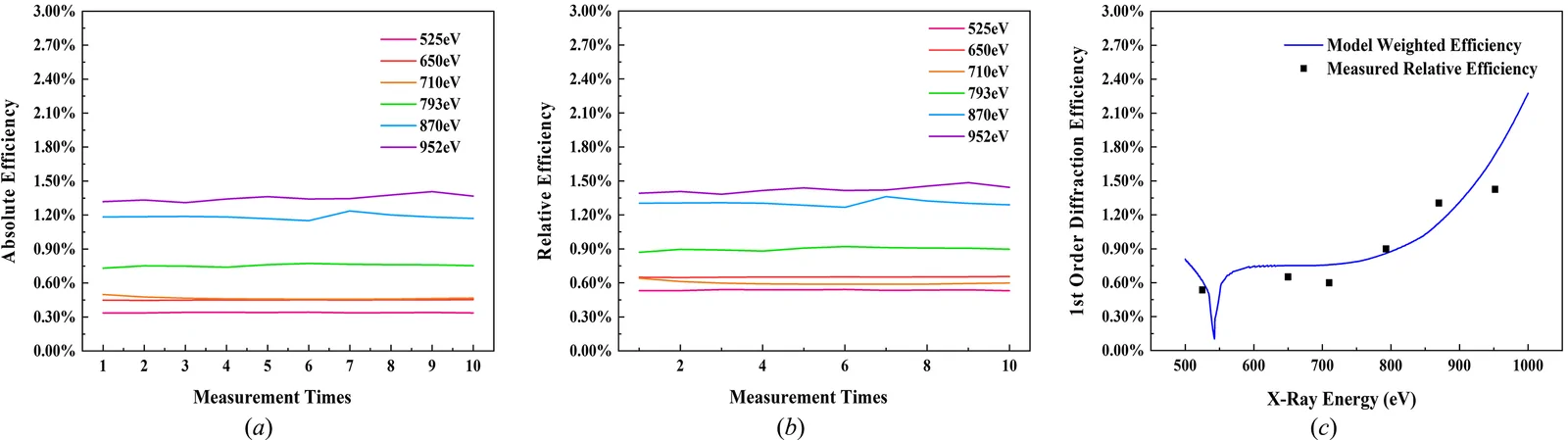
Diffraction efficiency measurements and theoretical benchmarking of the soft X-ray Fresnel zone plate. (*a*) Measured absolute diffraction efficiencies across the 525–952 eV photon energy range. (*b*) Measured relative diffraction efficiencies in the 525–952 eV range, excluding the absorption loss of the supporting membrane. (*c*) Overlay comparison of the measured relative diffraction efficiencies with the nominal RCWA predictions based on the designed geometry and tabulated optical constants. The values of 2.7%, 1.9%, and 2.1% discussed in the text denote the relative RMS fluctuations of the repeated absolute-efficiency measurements at 710, 870, and 952 eV, respectively. The *y*-axis values in all three panels are expressed as percentages.

We systematically obtained the absolute diffraction efficiencies, alongside the relative diffraction efficiencies that exclude supporting membrane attenuation, at six typical soft X-ray energy points: 525, 650, 710, 793, 870, and 952 eV. The results of the ten repeated measurements at each energy point are presented in Fig. 7 ▸. Benefiting from the beamline’s active closed-loop vibration suppression and real-time incident-flux normalization systems, the repeated measurements showed good short-term stability (Yao *et al.*, 2024 ▸; Jiao *et al.*, 2025 ▸). The repeatability of the efficiency measurements was evaluated using the relative RMS fluctuation,
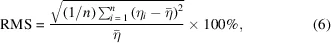
where η_*i*_ is the diffraction efficiency obtained from the *i*th repeated measurement, 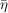 is the average efficiency, and *n* = 10. Using this definition, the relative RMS fluctuations of the absolute diffraction efficiencies at 710, 870, and 952 eV were 2.7%, 1.9%, and 2.1%, respectively. The relative RMS fluctuation of each repeated-measurement dataset remained below 3%, indicating good short-term measurement repeatability under the present beamline conditions. The full repeated-measurement sequence at each photon energy was of the order of minutes; therefore, these values characterize short-term repeatability during continuous measurements, rather than the absolute accuracy of the diffraction efficiency. The accuracy of the absolute diffraction efficiency is also affected by systematic factors in the normalization process, including the geometrical definition of the reference incident signal *I*_0_, dark-current subtraction, real-time incident-flux normalization, PMT linear response, and optical alignment. These factors were addressed through the aperture-defined *I*_0_ measurement geometry, dark-current correction, real-time incident-flux normalization using the BIMS, and PMT operation within the linear response range described above.

To evaluate the intrinsic optical focus capability of the device, the measured average relative diffraction efficiencies at each energy point were benchmarked against the nominal RCWA predictions based on the local grating approximation, designed geometry, and tabulated optical constants in Fig. 7 ▸(*c*). The results indicate that the measured relative efficiencies show an overall energy-dependent trend consistent with the nominal RCWA predictions over the measured 525–952 eV range. However, small residual deviations are still observed between the measured relative diffraction efficiencies and the nominal RCWA predictions. These deviations may arise from the combined differences between the actual FZP and the nominal model inputs, including the structural geometry and the effective material optical constants.

## 5. Conclusion

We developed a soft X-ray FZP with an outermost zone width of 12.5 nm, and systematically evaluated its imaging resolution and diffraction efficiency. Fabricated via a combination of electron-beam lithography and atomic layer deposition, the FZP achieved a high aspect ratio exceeding 6:1. Synchrotron X-ray microscopy tests demonstrated that the device can clearly resolve sub-15 nm edge features, achieving an FRC-estimated full-period spatial resolution of 13.0 nm based on the FRC criterion. Regarding diffraction efficiency metrology, we obtained both the absolute diffraction efficiency and the relative diffraction efficiency at six photon energies from 525 to 952 eV. The beamline’s active closed-loop system was used to suppress vibration-induced relative positional fluctuations (Yao *et al.*, 2024 ▸), whereas real-time incident-flux normalization was applied independently to the PMT signals (Jiao *et al.*, 2025 ▸). With these measures and rigorous optical alignment, the relative RMS fluctuation of ten consecutive measurements remained below 3%, indicating good short-term measurement repeatability under the present beamline conditions. Furthermore, benchmarking the measured relative diffraction efficiencies against the RCWA theoretical model reveals a highly consistent evolution trend across the measured soft X-ray band. These results provide a useful metrology reference under the present beamline conditions for developing advanced optical endstations at future high-brightness synchrotron radiation sources and X-ray free-electron laser facilities.
